# AI Chatbots in Answering Questions Related to Ocular Oncology: A Comparative Study Between DeepSeek v3, ChatGPT-4o, and Gemini 2.0

**DOI:** 10.7759/cureus.90773

**Published:** 2025-08-22

**Authors:** Deepsekhar Das, Atindra Narayan, Varsha Mishra, Lalit Takia, Sumit Grover, Avinav Bharati, Shrijith MB

**Affiliations:** 1 Ophthalmology, All India Institute of Medical Sciences, New Delhi, New Delhi, IND; 2 Medicine, All India Institute of Medical Sciences, New Delhi, New Delhi, IND; 3 Pediatrics, All India Institute of Medical Sciences, New Delhi, New Delhi, IND; 4 Medical Physics, All India Institute of Medical Sciences, New Delhi, New Delhi, IND; 5 Orthopedics, All India Institute of Medical Sciences, New Delhi, New Delhi, IND

**Keywords:** artificial intelligence, chatgpt 4o, deepseek v3, gemini 2.0, ocular oncology

## Abstract

Background

Artificial intelligence (AI) chatbots are increasingly used in healthcare for information dissemination and clinical decision support. However, their reliability and applicability in subspecialties such as ocular oncology remain largely unassessed. This study aimed to evaluate the accuracy, completeness, readability, and real-world utility of three prominent AI chatbots, ChatGPT-4o (OpenAI, San Francisco, California, USA), DeepSeek v3 (DeepSeek, Hangzhou, Zhejiang, China), and Gemini 2.0 (Google DeepMind, London, UK), in responding to clinically relevant questions related to ocular malignancies.

Methods

A cross-sectional observational study was conducted at a tertiary eye care institute in Northern India. Five clinical questions, covering key ocular oncologic conditions, were created and standardized by ocular oncology experts. These prompts were input into ChatGPT-4o, DeepSeek v3, and Gemini 2.0. Responses were independently evaluated using a structured proforma assessing correctness, completeness, readability (Flesch-Kincaid score, word count, sentence count), presence of irrelevant data, applicability in the Indian healthcare setting, and reliability. Data were analyzed using Kruskal-Wallis and ANOVA statistical tests.

Results

All three chatbots demonstrated comparable correctness scores (mean 3.4, SD 0.49). However, four out of five responses from each chatbot were deemed incomplete. DeepSeek v3 provided the most verbose and readable answers (mean 533.8 words; Flesch score 38.0), while ChatGPT-4o generated the shortest but more clinically reliable responses (mean reliability 3.2). Gemini 2.0 exhibited the greatest variability in length and structure. No irrelevant content was observed in any chatbot responses. Only 2/5 responses from ChatGPT-4o and 1/5 from each of the other two were directly applicable to Indian clinical practice.

Conclusion

While AI chatbots can offer factually accurate responses to ocular oncology-related queries, they often fall short in completeness and clinical applicability. ChatGPT-4o showed the most balanced performance, though regional customization and expert oversight remain essential. Current models are not yet suitable for unsupervised use in high-stakes clinical scenarios.

## Introduction

The discovery of artificial intelligence (AI) in the 21st century has opened a new dimension to the progress of scientific knowledge and research. AI basically consists of algorithms trained on various datasets to complete predefined tasks. Numerous technologies are emerging every day with the help of AI. AI is also evolving with time, making today's innovation obsolete tomorrow.

AI is slowly gaining more and more access and usage in human lives, and the healthcare sector is no exception. As days pass, more algorithms are being developed and implemented in medical sciences. The use cases in the field of medicine are endless, from the dissemination of patient information to use in diagnostic and therapeutic modalities [1].

AI is being used to process images of various pathology slides, microbiological slides, clinical images, radiological images, blood electrolyte parameters, and more. Although the technology is relatively new, it has huge potential for growth in the future [2-5].

AI chatbots are one of the most commonly used AI tools by many. There are various types of AI chatbots; a few can answer almost every question, whereas a few are developed in a way to answer questions regarding certain scientific or specific conditions. Popular chatbots include ChatGPT (OpenAI, San Francisco, California, USA), DeepSeek (DeepSeek, Hangzhou, Zhejiang, China), Gemini (Google DeepMind, London, UK), LLama (Meta AI, Menlo Park, California, USA), Copilot (GitHub Copilot, GitHub and OpenAI, California, USA), Consensus (Consensus, Boston, Massachusetts, USA), etc. [6,7].

These chatbots are trained on a huge database, which allows them to immediately answer any question that is asked of them. In the present day, patients are using these chatbots for seeking information regarding their diseases and management options, whereas medical students are using them for educational purposes and clearing doubts. Although the use of these chatbots is increasing every day, the information that is distributed by them mostly remains unchecked and unverified. There are limited studies on the authenticity and applicability of medical information obtained from these AI chatbots.

The authors noticed a lacuna in the credibility of the data provided by the chatbots on ocular cancer-related answers. To our knowledge, there were no studies assessing the readability and real-world applicability of answers on ocular and extraocular oncology that were generated by these AI tools in response to prompts.

The authors wanted to study the correctness, completeness, and whether the information provided by the AI chatbots can be used by patients or medical students.

## Materials and methods

A cross-sectional observational study was conducted at the All India Institute of Medical Sciences, New Delhi, a tertiary care eye institute in Northern India. The faculty members belonging to the ocular oncology department formulated a list of questions, which were asked as prompts in the available AI chat boxes.

Standardization of question prompts

It was ensured that every question contained data regarding age, gender, duration of symptoms, history of any systemic illness, history of any surgical treatment, history of any form of treatment the patient received in the past or was currently on, any positive family history, hematological, radiological, or any other information that the ocular oncologists deemed to be relevant in the planning of management. All questions contained a clinical diagnosis of a disease. The questions that were created included diagnosis of choroidal melanoma, ocular surface squamous neoplasia, retinoblastoma, sebaceous cell carcinoma, and basal cell carcinoma (Table 1).

**Table 1:** Questions related to ocular oncology USG: ultrasonography; CEMRI: contrast-enhanced magnetic resonance imaging; PET: positron emission tomography; UBM: ultrasound biomicroscopy

Questions	
1.	A 39-year-old male presented with complaints of diminution of vision of the right eye for the past five months, which was gradually progressive in nature. There is no history of any systemic illness or history of cancer in family members. Indirect ophthalmoscopy of the right eye revealed choroidal melanoma away from the fovea in the inferior quadrant. The other eye was within normal limits. A USG revealed a size of 5.32 X 9.12 mm lesion, which was confirmed on CEMRI head and orbit. A PET scan ruled out any systemic metastasis. Write a treatment plan for the condition.
2.	A 42-year-old non diabetic non hypertensive male presented with complaints of watering and a mass in the left eye for the past year. There is no history of any cancer in the family or any other major systemic illness. A slit lamp examination revealed a gelatinous lesion of size 10mm X 5mm involving the superior bulbar conjunctiva from 9 o'clock to 1 o'clock in the left eye. There was no corneal involvement. Impression cytology revealed features suggestive of ocular surface squamous neoplasia. There was no problem in the other eye. UBM revealed the lesion was limited to the ocular surface. Write a treatment for the condition.
3.	A 3-year-old male child was brought in with complaints of leukocoria of the right eye, as noted by the parents. On examination under anaesthesia, the right eye was diagnosed with group E retinoblastoma, left eye was diagnosed as multifocal group A retinoblastoma. The CEMRI head and orbit revealed no optic nerve head involvement or pineal gland tumour. Write a treatment for the patient.
4.	A 32-year-old pregnant female in her third trimester presented with a left lower eyelid mass that has been there for the past four months. On examination, the mass appeared to have a yellowish tinge on the palpebral conjunctiva. The size of the lesion was 15mm X 11mm. There was involvement of the eyelid margin. On incision biopsy, a diagnosis of sebaceous cell carcinoma was made. Hematological examinations were within normal limits. Write a treatment for the patient.
5.	A 68-year-old hypertensive male, farmer by profession, presented with a lower eyelid mass which has been there for two years. On examination, the mass appeared to be pigmented. The size of the lesion was 9mm X 15mm. The eyelid margin had an ulcer. CEMRI revealed a lesion in the lower eyelid that has not involved the orbital rim. Hematological tests revealed normocytic anaemia. An incision biopsy confirmed the diagnosis of basal cell carcinoma. Write a treatment for the patient.

The questions were evaluated by two faculty members of the same department to ascertain whether the mentioned criteria were met or not. On a specific day, after noting the date, the questions were put into the three AI chatbots, namely, DeepSeek v3, ChatGPT-4o, and Gemini 2.0. The answers by each chatbot were copied and pasted into Microsoft Word (Microsoft Corporation, Redmond, Washington, USA). Two independent faculties analyzed answers generated by AI chat boxes. 

The faculty designed a proforma, which was used for scoring each answer on the following parameters: correctness (absolutely correct/partially correct/wrong), assessment was made based on the most recent literature and evidence-based practice; completeness (complete/incomplete), assessment of whether the AI missed out on any data or not; and language and readability, the total number of words, sentences, and Flesch ease scores for readability [8].

Additional parameters used were irrelevant data (some/none), whether the chatbot stated irrelevant data; real-world applicability of the advice (word-for-word) in an Indian scenario (possible/not possible), if the treatment plan can be used word-for-word in an Indian scenario; and grade of reliability (not reliable/fairly reliable/highly reliable).

The data obtained was finally entered into MS Excel (Microsoft Corporation, Redmond, Washington, USA) and analyzed using IBM SPSS Statistics for Windows, Version 20 (Released 2011; IBM Corp., Armonk, New York, United States). Kruskal-Wallis tests were performed for correctness and reliability scores. One-way ANOVA and Levene’s test for homogeneity of variance were used for assessing language and readability parameters.

## Results

Correctness scores

All three models (ChatGPT-4o, DeepSeek v3, and Gemini 2.0) had a similar correctness score, averaging 3.4 with a standard deviation of 0.49. This suggests a consistent performance across the models in terms of accuracy (Tables 2-5).

**Table 2:** Scoring of answers by AI model ChatGPT-4o

Parameters	Answer 1	Answer 2	Answer 3	Answer 4	Answer 5
Correctness (1-4)	4	3	3	4	3
Completeness (incomplete/complete)	Incomplete	Incomplete	Incomplete	Complete	Incomplete
Language and readability (ease) (Flesch-Kincaid score)	31.37	45.13	19.66	26.72	34.42
Language and readability (total words)	275	308	277	304	271
Language and readability (total sentences)	32	31	22	29	23
Irrelevant data (NA/1-25%/25-50%, >50%)	NA	NA	NA	NA	NA
Additional relevant data (nil/some)	Some	Nil	Nil	Nil	Nil
Real-world applicability (word-to-word) in the Indian scenario	Not possible	Possible	Not possible	Possible	Not possible
Grade of reliability (1-4)	3	3	3	4	3

**Table 3:** Scoring of answers by AI model DeepSeek v3

Parameters	Answer 1	Answer 2	Answer 3	Answer 4	Answer 5
Correctness (1-4)	4	3	3	4	3
Completeness (incomplete/complete)	Complete	Incomplete	Incomplete	Incomplete	Incomplete
Language and readability (ease) (Flesch-Kincaid score)	33.34	42.65	36.78	35.22	42.04
Language and readability (total sentences)	54	44	54	52	54
Language and readability (total words)	481	476	608	567	537
Irrelevant data (NA/1-25%/25-50%, >50%)	NA	NA	NA	NA	NA
Additional relevant data (nil/some)	Some	Nil	Nil	Nil	Nil
Real-world applicability (word-to-word) in the Indian scenario	Not possible	Possible	Not possible	Not possible	Not possible
Grade of reliability (1-4)	3	3	3	2	3

**Table 4:** Scoring of answers by AI model Gemini 2.0

Parameters	Answer 1	Answer 2	Answer 3	Answer 4	Answer 5
Correctness (1-4)	4	3	3	4	3
Completeness (incomplete/complete)	Complete	Incomplete	Incomplete	Incomplete	Incomplete
Language and readability (ease) (Flesch-Kincaid score)	40.58	42.50	44.62	26.43	28.67
Language and readability (total sentences)	21	19	23	59	45
Language and readability (total words)	425	322	346	636	598
Irrelevant data (NA/1-25%/25-50%, >50%)	NA	NA	NA	NA	NA
Additional relevant data (nil/some)	Some	Nil	Nil	Nil	Nil
Real-world applicability (word-to-word) in the Indian scenario	Not possible	Possible	Not possible	Not possible	Not possible
Grade of reliability (1-4)	3	3	3	3	3

**Table 5:** Consolidated results from ChatGPT-4o, DeepSeek v3, and Gemini 2.0

ChatGPT-4o						DeepSeek v3					Gemini 2.0				
Parameters	Answer 1	Answer 2	Answer 3	Answer 4	Answer 5	Answer 1	Answer 2	Answer 3	Answer 4	Answer 5	Answer 1	Answer 2	Answer 3	Answer 4	Answer 5
Correctness (1-4)	4	3	3	4	3	4	3	3	4	3	4	3	3	4	3
Completeness (incomplete/complete)	Incomplete	Incomplete	Incomplete	Complete	Incomplete	Complete	Incomplete	Incomplete	Incomplete	Incomplete	Complete	Incomplete	Incomplete	Incomplete	Incomplete
Language and readability (ease) (Flesch-Kincaid score)	31.37	45.13	19.66	26.72	34.4	33.34	42.65	36.78	35.22	42.04	40.58	42.5	44.62	26.43	28.67
Language and readability (total words)	275	308	277	304	271	54	44	54	52	54	21	19	23	59	45
Language and readability (total sentences)	32	31	22	29	23	481	476	608	567	537	425	322	346	636	598
Irrelevant data (NA/1-25%/25-50%, >50%)	NA	NA	NA	NA	NA	NA	NA	NA	NA	NA	NA	NA	NA	NA	NA
Additional relevant data (nil/some)	Some	Nil	Nil	Nil	Nil	Some	Nil	Nil	Nil	Nil	Some	Nil	Nil	Nil	Nil
Real-world applicability (word-to-word) in the Indian scenario	Not possible	Possible	Not possible	Possible	Not possible	Not possible	Possible	Not possible	Not possible	Not possible	Not possible	Possible	Not possible	Not possible	Not possible
Grade of reliability (1-4)	3	3	3	4	3	3	3	3	2	3	3	3	3	3	3

Completeness

Four out of five answers generated by all three chatbots were incomplete, due to a lack of data, which is routinely used by the authors for the treatment of diseased conditions, as mentioned.

Readability scores (Flesch-Kincaid ease)

**Figure 1:**
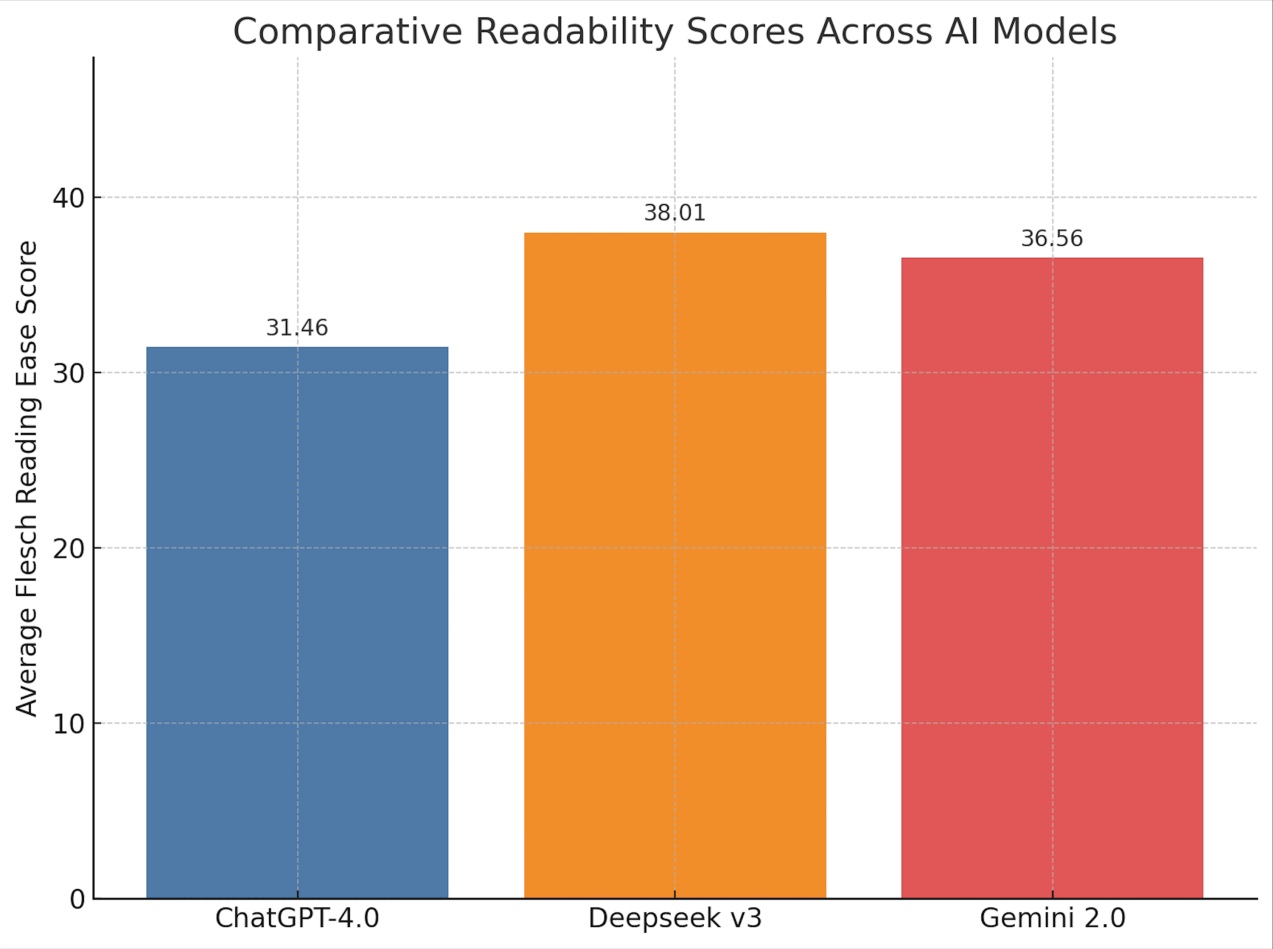
Comparative readability scores across AI models

DeepSeek v3 had the highest readability score (38.01) with the least variation, suggesting more consistently readable answers. Gemini 2.0 showed the greatest variation (std dev 7.5), indicating inconsistency in sentence complexity (Figure 1).

Total words

DeepSeek generated the most extensive answers (mean 533.8 words), while ChatGPT-4o had the shortest (287 words). Gemini 2.0 had the highest variation in word count (std dev 128.9), suggesting inconsistency in response lengths.

Total sentences

DeepSeek used the most sentences per response (mean 51.6 sentences), while ChatGPT-4o had the fewest (27.4). Gemini 2.0 had a wide variation (std dev 15.8), indicating fluctuation in sentence structure.

Irrelevant data

There was no irrelevant data generated by any of the AI chatbots while answering the queries. Real-world word-to-word applicability in the Indian scenario: 2/5 answers generated by ChatGPT-4o, and 1/5 answers generated by DeepSeek v3 and Gemini 2.0 were found to be possible for word-to-word use in the Indian scenario.

Reliability scores

ChatGPT-4o had the highest reliability mean (3.2), followed by Gemini 2.0 (3.0) and DeepSeek v3 (2.8). DeepSeek v3 showed a slight inconsistency in reliability, indicated by its higher standard deviation (0.4). 

For correctness and grade of reliability, we have used weighted Cohen's kappa, which showed almost perfect agreement for both. We calculated Cohen's kappa for completeness/irrelevance data and the real-world applicability of the advice (word-for-word) in the Indian scenario. There was perfect agreement (k=1).

## Discussion

The convergence of AI and healthcare has ushered in a transformative era, marked by the emergence of sophisticated large language models (LLMs) such as ChatGPT-4o, DeepSeek v3, and Gemini 2.0. These models are designed to generate human-like responses and have shown promise across a wide spectrum of medical applications, including patient education, triage, medical writing, and clinical decision support [9]. However, their adoption in high-stakes, domain-specific specialties such as ocular oncology remains underexplored and necessitates rigorous evaluation before integration into clinical workflows.

Ocular oncology: a high-stakes specialty

Ocular oncology is a specialized branch of ophthalmology focusing on the diagnosis and management of benign and malignant neoplasms involving the eye, orbit, and ocular adnexa. Conditions such as retinoblastoma, uveal melanoma, ocular surface squamous neoplasia (OSSN), sebaceous gland carcinoma (SGC), and basal cell carcinoma (BCC) pose unique diagnostic and therapeutic challenges. Management decisions often depend on precise histopathological diagnoses, imaging findings (ultrasound, MRI, optical coherence tomography (OCT)), molecular markers, patient demographics, and region-specific treatment availability [10]. Currently, the form of medicine that is being most widely accepted and routinely practiced worldwide is evidence-based medicine. However, if the credibility of the evidence is lacking, then the purpose as a whole is flawed. The sources of information, especially when dealing with medical and healthcare queries and prompts, must be backed up and supported by authentic sources and literature. In the forehand, certain information is only obtained through strenuous training and may be subject to proprietary limitations. There remains a grey line as to whether the LLMs can have access to this confidential data. This again can possibly be attributed to the reduced completeness and correctness scores. Therefore, superficial or overly generalized information from AI systems can lead to inappropriate guidance, potentially causing patient harm or clinician misdirection.

The current study critically evaluates three prominent LLM-based chatbots, ChatGPT-4o, DeepSeek v3, and Gemini 2.0, for their accuracy, completeness, readability, contextual relevance, and alignment with Indian healthcare standards in answering ocular oncology-related queries. This approach serves to fill a critical knowledge gap regarding the trustworthiness and clinical applicability of generative AI tools in a highly specialized and sensitive domain.

Accuracy versus clinical relevance

While all three chatbots exhibited comparable correctness scores, this metric alone does not sufficiently capture the clinical reliability of the responses. A significant observation was that approximately 80% of responses lacked essential details such as differential diagnoses, staging criteria, or standard-of-care therapeutic options. Such omissions could misguide users seeking informed medical decisions.

DeepSeek v3, although generating the most elaborate responses, often lacked clinical precision. This echoes findings from previous studies where verbosity was inversely associated with medical utility [11]. In contrast, ChatGPT-4o, with its concise yet focused answers, displayed higher fidelity to evidence-based guidelines and better contextual relevance, especially in questions that invoked Indian treatment frameworks. This balance between brevity and precision is crucial in clinical communication, where overcomplicated responses may confuse patients or non-specialist providers, while overly terse replies may omit key information [12].

Readability and accessibility

From a readability standpoint, DeepSeek v3 achieved the highest Flesch-Kincaid readability score, suggesting its responses may be more accessible to laypersons. While high readability is desirable in patient education, it should not come at the expense of factual accuracy or clinical relevance. On the other hand, ChatGPT-4o struck a better balance by producing responses that were both moderately readable and clinically valid. These findings are consistent with prior research, which emphasized the importance of optimizing LLM outputs for target audiences, clinicians, patients, or students, based on language complexity and domain relevance [6].

Regional relevance and localization challenges

One of the most critical limitations identified was the lack of regional specificity across all three models. Only a minority of responses incorporated Indian clinical protocols or addressed country-specific realities such as access to brachytherapy, limitations of public healthcare infrastructure, or the use of cost-effective diagnostics. Localization of AI tools is essential, particularly in low- and middle-income countries (LMICs), where variations in resource availability, diagnostic workflows, and epidemiological patterns require tailored medical recommendations [13].

Globally trained LLMs tend to generalize based on predominant datasets from high-income countries, leading to a mismatch when applied in different healthcare systems. For example, the management approach to retinoblastoma in India may include chemoreduction and focal therapies prioritizing eye salvage, while Western protocols might emphasize enucleation earlier in the disease course due to earlier detection. These contextual nuances are often absent in current chatbot outputs. Therefore, for AI systems to be clinically viable, especially in LMICs, integration with region-specific guidelines like those of the All India Ophthalmological Society (AIOS) or the Indian Council of Medical Research (ICMR) becomes imperative [14].

Response structure and consistency

Another important finding was the inconsistency in response structure, particularly evident in Gemini 2.0. Unstable formatting, variable tone, and occasional deviation from the question posed reduce the reliability and professionalism expected of a medical advisory tool. This inconsistency may stem from prompt interpretation variability or inadequate fine-tuning on healthcare-specific datasets, a challenge previously reported in studies assessing chatbot responses in oncology and cardiology [15-17].

ChatGPT-4o demonstrated greater uniformity in both structure and tone, improving trust and usability. Uniform formatting and transparent citation of medical sources, where applicable, could further enhance the credibility and adoption of these platforms.

Comparison with previous research

Our findings resonate with previous research highlighting the limitations of LLMs in specialized medical domains. Sandmann et al. (2024) conducted a study on ChatGPT’s use in oncology and concluded that although the model could answer basic questions adequately, it faltered in complex scenarios lacking contextual specificity, often providing outdated or generic treatment recommendations [6]. Similarly, Rossettini et al. (2023) found wide variability in chatbot performance on standard physiotherapy assessment tools, emphasizing the risk of misleading information in patient self-care and rehabilitation [7]. In line with our evaluation of chatbot accuracy and completeness in ophthalmic oncology, Sallam et al. (2025) recently benchmarked Chinese genAI models such as DeepSeek and Qwen (Alibaba Cloud, Hangzhou, China) against ChatGPT-4o, reporting superior performance in completeness, factual accuracy, and relevance in both English and Arabic ophthalmology queries [18]. 

Furthermore, a recent article by Kung et al. tested ChatGPT on the United States Medical Licensing Examination (USMLE) and reported a pass rate near the threshold but warned against deploying the model for clinical decision-making without oversight due to hallucinations and contextual gaps [19].

Implications for clinical practice and future development

The utility of AI chatbots in ocular oncology currently lies more in supplementary education and patient awareness than in independent clinical decision-making. These concerns parallel the findings of a broader strengths, weaknesses, opportunities, and threats (SWOT) analysis by Sallam et al. (2025), which underscored that safe AI adoption in clinical practice requires region-specific customization, rigorous validation, and continued human oversight, particularly in high-stakes domains [20]. To bridge the gap between potential and practice, several enhancements are necessary: fine-tuning with domain-specific datasets by incorporating large, peer-reviewed datasets focused on ocular oncology to enhance accuracy; integration with real-time medical databases by linking chatbots to continuously updated resources like PubMed, AIOS guidelines, and ICMR protocols to improve temporal relevance; localization algorithms by embedding socio-cultural, economic, and infrastructural variables that could personalize advice for regional settings; and human-in-the-loop (HITL) validation by establishing a workflow where AI responses are reviewed by qualified professionals before dissemination to ensure safety and credibility.

Moreover, interdisciplinary collaboration between AI developers, ophthalmologists, and healthcare policymakers will be key to developing ethically robust, clinically useful, and culturally contextualized AI tools. The sphere of AI chatbots is expanding exponentially, with newer upgrades coming every month. Although in our study we found that these chatbots at present cannot be used with a high degree of confidence in obtaining correct answers or be used in Indian scenarios, keeping the decent developments in mind, we are not too far away from that day when they can be actually implemented. Various faster and better AI LLMs, like Grok4 (X.AI Corp., California, USA) and Perplexity (Aravind Srinivas, California, USA), have emerged, which warrants the need for newer studies for benchmarking the reliability of the information provided by these chatbots.

Limitations

Single-day/single-run responses: As the study was conducted in a single day, it does not allow the assessment of the continuous upgradation and evolution that is ongoing regularly in the various LLM models. Using a single prompt, although it allowed us to compare the responses amongst various chatbots, is again a limitation, as even a slight, minor tweaking in the prompt could generate a completely different answer. In order to maintain uniformity, we did not ask or add any follow-up prompts to enhance the answers, which we acknowledge as another limitation.

In order to deal with the limitations of the current study, there is a requirement for larger follow-up studies in the future with a larger set of questions, variables, and different formats of data (images, videos, and multimedia files). There is also a need to assess the response of the chatbots to adversarial and ambiguous queries, which will further enhance our understanding of these LLM models, thereby increasing the ease of their usage.

## Conclusions

This comparative analysis highlights the strengths and limitations of AI chatbots in addressing ocular oncology queries. While correctness scores were similar across ChatGPT-4o, DeepSeek v3, and Gemini 2.0, major gaps were observed in the completeness of clinical responses. DeepSeek v3 offered the most extensive and readable content, yet scored lowest on reliability, indicating that verbosity does not guarantee accuracy or clinical soundness. ChatGPT-4o, despite producing more concise answers, was relatively more reliable and had better alignment with Indian clinical practices. Gemini 2.0 demonstrated fluctuating performance across most parameters, reflecting an inconsistency in both structure and clarity.

Notably, all models successfully avoided irrelevant data, showing that their contextual understanding is well-calibrated. However, the limited real-world applicability of responses in the Indian healthcare setting reveals a critical area for improvement. AI models must evolve to incorporate region-specific guidelines and treatment norms to truly support medical decision-making. In conclusion, while AI chatbots show promise in supplementing medical education and information retrieval, they are not yet ready to independently guide patient care in complex specialties like ocular oncology. Human oversight remains essential, and customization for regional contexts will be a key step toward clinical relevance and integration.
